# Screened Realities: Exploring the Intensity and Portrayal of Youth Suicide in Films

**DOI:** 10.7759/cureus.92386

**Published:** 2025-09-15

**Authors:** Farhad Huseynov, Deryanur Celik, Zheala Qayyum

**Affiliations:** 1 Psychiatry, Bursa Uludağ University, Bursa, TUR; 2 Infectious Diseases and Clinical Microbiology, Prof. Dr. Cemil Tascıoglu City Hospital, Istanbul, TUR; 3 Psychiatry and Behavioral Sciences, Boston Children's Hospital, Harvard Medical School, Boston, USA

**Keywords:** films, media, movies, suicide, youth

## Abstract

Introduction

Suicide remains one of the leading causes of death among youth aged 15-29 years, and how it is portrayed in the media can influence public perception.

Methods

This study aimed to evaluate the intensity of youth suicide portrayals in movies, focusing on whether these portrayals reflect real-life risk factors and offer responsible messaging. We analyzed 11 films using a mixed-method approach: the Movies and Video: Identification and Emotions in reaction to Suicide (MoVIES) scale was used to assess the intensity of scenes depicting suicide, and the Substance Abuse and Mental Health Services Administration (SAMHSA) Suicide Assessment Five-step Evaluation and Triage (SAFE-T) tool helped identify risk and protective factors that were shown in the movies. We also conducted content analysis to explore emerging themes.

Results

The results showed that while most movies included realistic stressors, none portrayed a family history of suicide, psychiatric hospitalization, or preventive interventions. A few films stood out with higher MoVIES scores and lacked content warnings, which could pose a risk for vulnerable audiences.

Conclusions

While not every portrayal leads to harm, it is clear that the intensity of suicide depiction without guidance or support can do more damage than good. We believe there is a need for more collaboration between filmmakers and mental health professionals to ensure that sensitive topics like suicide are portrayed in a way that can open a dialogue and enhance awareness.

## Introduction

Suicide is a multifaceted public health problem, leading to more than 720,000 deaths every year [1]. Accounting for approximately 1% to 1.4% of all deaths, suicide is still one of the leading causes of death among people aged 15-29 years [2]. Among those aged 15-19 years specifically, it accounted for 9.1% of all deaths, highlighting its significant impact on younger populations [3]. This highlights unique dynamics and draws special attention to this age group [4]. Some factors that make this age group prone to attempting suicide include a high prevalence of mental health disorders, social and academic pressures stemming from school, relationships, and societal expectations, as well as challenging family dynamics [5]. A developing teenage brain further compounds these risks, as the prefrontal cortex, responsible for analyzing risks, regulating emotions, and controlling impulses, is still undergoing myelination, which continues into the mid-20s [6]. Without its complete regulatory functions, teenagers are more prone to reacting impulsively to distressing situations, such as a breakup, social rejection, or academic failure [7]. This heightened impulsivity, combined with social isolation exacerbated by loneliness or bullying, further contributes to this vulnerability [8]. The desire for validation and inclusion, often fueled by social media, can also be influenced by the narratives presented in popular media, such as movies, where portrayals of suicide may be perceived as dramatic or a means to get noticed or gain attention [9].

Given that adolescents are avid movie consumers and the film industry generates massive revenues, suicide portrayal is a significant concern for this vulnerable age group [10]. With the rise of social media, these portrayals are no longer confined to the big screen. A single scene can be clipped into short-form videos (called reels), gaining millions of views within hours. Unlike traditional media, where content had temporal and geographic constraints, these clips circulate on the internet without boundaries, unrestricted by time or location. The term “Werther effect” has been used to explain that sensationalized media coverage can lead to increased suicide rates among young people [11]. This effect is especially concerning in the context of popular media, where dramatic portrayals of suicide can inadvertently encourage imitating behaviors [12].

To ensure safer media coverage and prevent suicide, guidelines for reporting suicides in films have been developed by the World Health Organization (WHO) and various national agencies [13]. The booklet guides filmmakers and editors by listing reporting characteristics that maximize positive impact, prevent imitation effects, and promote help-seeking and hope. For example, they recommend avoiding depictions of the suicide act or the specific method used.

The aim of the current study is to explore how suicide is portrayed in movies, with a particular focus on content targeting or affecting individuals aged 15-30 years. We evaluated both the intensity and nature of these portrayals, quantitatively and qualitatively, to assess whether they accurately reflect the real-life complexity of suicide.

## Materials and methods

We used a mixed-method approach, incorporating both qualitative and quantitative data. The study was conducted between January and March 2025. We conducted content analysis to collect the qualitative data, and used the Movies and Video: Identification and Emotions in reaction to Suicide (MoVIES) scale and the Substance Abuse and Mental Health Services Administration (SAMHSA) Suicide Assessment Five-step Evaluation and Triage (SAFE-T) assessment for quantitative data [14,15].

In our study, we utilized the MoVIES scale, which was created for scoring movies portraying suicidal behaviors [14]. The MoVIES scale is validated and reliable. It is designed to assign a numerical score to suicide-related content in video productions in terms of their potential effect on the viewer. It includes 41 items, each scored as either 0 (absent) or 1 (present). Higher total scores indicate more intense and potentially triggering portrayals of suicide. Two raters were used because this is standard in inter-rater reliability studies as it balances rigor, accuracy, and feasibility. For each film, the entire movie was viewed by both raters, ensuring that all relevant context and narrative elements were included in the analysis. Two raters (F.H. and D.C.) independently filled out the other after watching each movie. Before starting the actual study, two raters did a trial run using a movie that included a suicide scene but was not part of this study. This allowed them to reach inter-rater consensus and ensure they were interpreting and rating items consistently. After completing all films, we calculated the inter-rater reliability of each item by measuring the corresponding Cohen’s kappa with statistical significance defined as p < 0.05. The agreement of items with a kappa score lower than 0.6 was considered insufficient. Quantitative data were analyzed using descriptive statistics, specifically mean scores of MoVIES and SAFE-T ratings across the two raters. We conducted the analysis using R 4.4.3 (R Foundation for Statistical Computing, Vienna, Austria) [16].

We analyzed both the overall scores of each movie individually and the performance of each item across the sample on the SAFE-T and MoVIES scales. For the qualitative component, we used an inductive content analysis approach, guided by Krippendorff’s methodology for content analysis [17]. Two raters (F.H. and D.C.) independently reviewed the films and extracted text passages, character interactions, and narrative elements relevant to suicide. These excerpts were coded manually, without software, and grouped into preliminary categories. Codes were compared and refined through iterative discussion until consensus was reached. From these categories, broader themes (e.g., stigma, family pressure, and absence of support) were derived. Disagreements were resolved through joint review of the material until full agreement was achieved. This process allowed us to identify recurring patterns and narrative meanings that complemented the quantitative findings.

SAFE-T is a suicide assessment tool developed from the American Psychiatric Association Practice Guidelines for the Assessment and Treatment of Patients with Suicidal Behaviors [15]. It aims to determine a patient's risk level through a five-step evaluation. It includes seven risk factors and two protective factors. Although the tool itself is not originally designed to be scored in this way, we used it as a reference framework and assigned scores of 0 (not present) or 1 (present) for each item. Similarly, two raters (F.H. and D.C.) independently filled out the other after watching each movie. This allowed us to get a sense of how comprehensively the movies portrayed or hinted at these suicide-related factors and provided additional quantitative data for comparison. Any scoring discrepancies were resolved through discussion until consensus was reached.

No ethical approval was required for this study, as it involved only the analysis of publicly available films and did not include human participants, identifiable data, or animal subjects.

Sample

We used open movie search platforms such as IMDB and Letterboxd by using keywords including “suicide”, “teen”, and “adolescent”. The inclusion criteria were the presence of at least one scene depicting a suicide attempt or death by suicide involving a character aged 15-30 years. The exclusion criterion was the absence of such a scene within this specified age group. Since the MoVIES scale scores the suicide scenes, films meeting the inclusion criterion were selected. We included 10 movies from the initial search and excluded one for not depicting a direct suicidal scene. Subsequently, we conducted a deeper search to increase the sample, which led to the addition of two more relevant movies. All available movies identified with these keywords and containing relevant scenes were included, regardless of their rating, popularity, or awards, to ensure the sample was as comprehensive as possible based on the search results. To maintain anonymity and comply with journal guidelines regarding potential identifiers, the names of movies have been excluded and replaced by numbers (Movie 1 to Movie 11) throughout the manuscript and tables.

## Results

Tables 1, 2 present the comprehensive findings from our analysis of 11 movies depicting suicide. The first table includes MoVIES score, main themes from the perspective of characters who attempted suicide, and representative quotes for each movie. The second table provides the SAFE-T score.

**Table 1:** The MoVIES scale score reflects the intensity of suicide portrayal (higher = more intense). The scores are the average of two independent raters. MoVIES: Movies and Video: Identification and Emotions in reaction to Suicide.

Movie name	Country and duration	Total MoVIES scale score	Main theme from the perspective of a character who attempted suicide
1	United States, 2 hours and 8 minutes	25	A teenage boy being trapped by parental expectations, with a desperate need for autonomy and self-expression
2	United States, 1 hour and 37 minutes	13.5	The five daughters rebel against the intrusive repression imposed by their family
3	United States, 2 hours and 7 minutes	14	A young woman diagnosed with a psychiatric condition is discharged from the hospital while continuing to endure abuse from her father
4	France, 1 hour and 55 minutes	17	A sibling’s desperate act blurs love, innocence, and self-destruction when reality threatens their fragile world
5	Australia, 1 hour and 31 minutes	25.5	An adolescent overwhelmed by hidden trauma and isolation
6	United States, 2 hours	16.5	A boy struggles with rejection from his mother due to his sexual orientation
7	Norway, 1 hour and 35 minutes	13	A young adult receiving psychiatric treatment for substance use disorder grapples with persistent stigma
8	United States, 1 hour and 38 minutes	24.5	A high school girl, neglected and bullied, feels isolated both at school and at home
9	Belgium, 1 hour and 35 minutes	12	A working-class mother battles severe depression, worsened by job insecurity and social isolation
10	United States, 1 hour and 55 minutes	12	A young woman faces unaddressed mental health challenges alongside growing professional frustrations
11	United States, 2 hours and 17 minutes	14	A boy in desperate need of connection and belonging is devastated after his new friend dies by suicide

**Table 2:** The SAFE-T score shows the total number of suicide risk and protective factors depicted. SAFE-T: Suicide Assessment Five-step Evaluation and Triage; rf: risk factor; pf: protective factor.

Movie name	SAFE-T scale score	Risk factors according to the SAFE-T scale	Protective factors according to the SAFE-T scale
1	4 (3 rf, 1 pf)	Hopelessness, triggering events leading to despair, access to firearms	Social support
2	2 (2 rf, 0 pf)	Hopelessness, triggering events leading to despair	
3	3 (3 rf, 0 pf)	Current psychiatric disorder, triggering events leading to humiliation, change in treatment	
4	1 (1 rf, 0 pf)	Triggering events leading to humiliation	
5	1 (1 rf, 0 pf)	Triggering events leading to despair	
6	3 (3 rf, 0 pf)	Previous suicide attempt, anhedonia, hopelessness, triggering events leading to humiliation	
7	6 (4 rf, 2 pf)	Previous suicide attempt, substance use disorder, hopelessness, shame, change in treatment	Ability to cope with stress, social support
8	2 (2 rf, 0 pf)	Anhedonia, hopelessness, triggering events leading to humiliation	
9	4 (3 rf, 1 pf)	Major depressive disorder, anhedonia, anxiety/panic, hopelessness, triggering events leading to humiliation	Responsibility to children, social support
10	2 (2 rf, 0 pf)	Triggering events leading to humiliation, access to firearms	
11	3 (3 rf, 0 pf)	Current psychiatric disorder, anxiety/panic, hopelessness, triggering events leading to despair	

The overall Cohen’s kappa for the MoVIES scale data was 0.6849 (95% CI: 0.558-0.790, p < 0.001), indicating substantial agreement between raters, which is highly significant beyond chance. For 27 items, the kappa values were above 0.6; six items were below this threshold, and for eight items, Cohen’s kappa could not be calculated because all scores were identical across both raters. These missing kappa values occurred due to complete agreement on those items, meaning the raters fully agreed in all cases.

Quantitative results

Precipitants/stressors/interpersonal risk factors were present throughout all the movies. None of them presented in a trivial way, nor was suicide ever shown as a solution.

Family history of suicide or psychiatric hospitalization was completely absent from all the movies.

Another major point, according to scale, was the absence of interventions that could potentially have contributed to preventing suicide in all movies.

There is a noticeable clustering of movies in the 12-17 range (n = 8); however, three films (*Dead Poets Society*, *2:37*, and *Detachment*) scored significantly higher in the 24-26 range in the MoVIES scale. These movies are clearly more emotionally intense and potentially distressing, particularly for vulnerable populations. Notably, none of them included content warnings regarding their strong portrayals of suicide.

The highest SAFE-T risk/protective factor score was 6 (out of 9) in Movie 7, which also had a relatively low MoVIES score (13). A high SAFE-T score indicates the presence of multiple identifiable risk and protective factors, making this movie useful for teaching purposes, especially in recognizing warning signs. It could facilitate understanding how to approach individuals at risk, or even how not to.

Qualitative results

Out of the 11 movies, four featured characters with a psychiatric diagnosis (major depressive disorder, substance use disorder, borderline personality disorder, generalized anxiety disorder). Two of these characters faced clear signs of stigma, which, unfortunately, reflect real-world issues. In Movie 7, the suicidal character receives cruel jokes about his addiction, and even his sister refuses to see him. In Movie 9, the character is at risk of losing her job due to receiving psychiatric treatment. Her boss expresses concern about her ability to manage the same workload, reflecting workplace stigma.

In three movies, the reason behind suicide was primarily rooted in family pressure, though for different reasons. In Movie 1, the pressure stemmed from academic expectations. In Movie 2, unbearable pressure related to social constraints from parents was the leading cause for the suicides of five daughters. In Movie 6, homosexuality was the main domain of conflict within the family, specifically with the mother. According to the Interpersonal Theory of Suicide, thwarted belongingness, such as family rejection due to homosexuality, can increase suicide risk by creating profound loneliness and disconnection [18]. Research also shows that films effectively communicate these experiences to viewers, allowing them to recognize and empathize with depicted loneliness or exclusion, which may impact their own perceptions of suicide risk. These stories emphasize the absence of family support; rather, disapproval or rejection from family has a major impact on the emotional well-being of the youth. The feeling of not being accepted leads to an overwhelming sense of emptiness and helplessness, making young people feel as if there is no way out, as if it is “the end of the world”.

In Movie 5, we see the problems and backgrounds of six high school students, but it is the character who is least noticed that dies by suicide. Through a few brief scenes, we get some sense that she was feeling alone, but there was no apparent reason, leaving the other six youths confused as well. This highlights the complexity of suicide. “Sometimes you get so wrapped up in your own problems that you just don’t notice anybody else”.

## Discussion

Studies have shown that a family history of completed suicide significantly increases suicide risk [19]. This elevated risk most likely reflects a combination of factors, including shared genetic predispositions (such as impulsivity or mood disorders), the presence of psychiatric illness among family members, exposure to maladaptive coping, direct modeling of suicidal behavior, and sometimes adverse family environments. For example, one recent study found that the risk of a fatal suicide attempt was increased threefold in those left behind after suicide compared to those who lost loved ones to other causes [20]. Despite this, none of the movies in our sample depicted a family history of suicide or psychiatric hospitalization, highlighting a potential gap in realistic portrayals of risk factors.

Community outreach, crisis intervention, or providing access to crisis hotlines could have been helpful for triggered audiences [21]. However, our results indicate that none of the movies included depictions of preventive interventions or resources, underscoring a missed opportunity to model help-seeking and support for at-risk viewers.

Our findings also highlight how stigma in the workplace was a central theme in one of the films. In this case, a character risked losing her job due to psychiatric treatment, and her employer expressed doubts about her ability to manage the same workload. Such portrayals mirror real-world toxic workplace cultures, where fear of professional and socioeconomic consequences can discourage individuals from seeking mental healthcare.

The use of documentaries such as “The Bridge” in medical school curricula has been perceived as a useful tool for understanding the complexity of suicide, suggesting that media can play a constructive educational role when handled sensitively [22]. Similarly, “Oslo, August 31st” from our sample may be valuable in educational settings. With its relatively low MoVIES score but the highest SAFE-T risk/protective factor score, it effectively illustrates warning signs and risk factors. This film could help teach students how to recognize individuals at risk and discuss appropriate approaches, supporting the use of media to enhance suicide prevention education.

There is no declared consensus about the impact of media on actual suicidality. In a systematic review that included 56 studies, it was shown that most studies support an association between media reporting and suicide [23]. However, the main challenge in establishing a clear association between media reporting and suicide lies in demonstrating exact causality, as numerous confounding factors, such as individual vulnerabilities, social influences, and broader environmental conditions, can also contribute to suicidal behaviors.

*13 Reasons Why* is one of the most recent main social media content that drew considerable attention on social media itself and among health professionals. The creators of *13 Reasons Why* intended to inform and educate viewers about bullying, depression, sexual assault, and suicide through a powerful and emotionally engaging narrative, hoping to spark a wider conversation and make these issues appear disturbing and unappealing. Despite these good intentions, which align with the goals of mental health professionals, the series was criticized for its approach. This popular series has been widely viewed, to the point that even the authors took caution against exposing children and adolescents to the series [12,24]. This, in turn, potentially increases the risk of imitative behaviors among adolescents seeking belonging or self-expression [25]. Multiple research studies indicate that media portrayals of suicide can have a profound impact on adolescents, particularly vulnerable youth with psychiatric illness or stressful life events [26]. Professionals warn that youth, especially those with pre-existing mental health conditions, are particularly susceptible to suicide contagion when the media normalizes, sensationalizes, or glorifies suicide [26]. After the release of this show, cumulative internet searches for suicide-related terms increased. While searches for “suicide prevention,” “teen suicide,” and “suicide hotline” rose, so did the searches for “how to commit suicide” and similar harmful phrases [27]. This dual impact highlights the complex influence of media portrayals on public mental health. In our sample, the clustering of emotionally intense films in the higher MoVIES score range, without content warnings, raises concerns about the potential for such portrayals to impact vulnerable viewers, which does not align with WHO guidelines emphasizing the importance of content warnings and responsible messaging. This highlights a missed opportunity for modeling help-seeking behaviors, and a need for responsible media portrayals that inform without causing harm.

Nevertheless, it is possible to portray suicide in a way that cultivates hope by increasing awareness of available supports for those who struggle with suicidal thoughts or behaviors. The importance of freedom of expression and concerns about censorship by media creators are understandable. However, there is a fine line between expressing feelings with the aim of helping people and inadvertently causing harm, even retraumatizing. Although concerns about censorship and loss of creative license may dampen enthusiasm in the entertainment industry, there is an urgent need for professionals to be involved with media communities to ensure that no harm is done [28].

One suicide themed movie was excluded from our study because it does not include any suicide scenes. It is only mentioned that the character died by suicide, and it focuses on how people process this news. If evaluated using the MoVIES scale, it would receive a score of 0. Whether the directors or screenwriters were aware of the WHO guidelines during production cannot be fully determined. However, it is clear that they intentionally avoided showing explicit suicide scenes or even mentioning the means of suicide. We could even go further and say that it serves as an excellent example of how suicide scenes should be portrayed and handled.

In summary, our analysis of 11 films revealed that while realistic stressors were commonly portrayed, key risk factors such as family history of suicide and psychiatric hospitalization were consistently absent. Furthermore, preventive interventions were never depicted, and several films with high MoVIES scores lacked content warnings, raising concerns about their potential impact on vulnerable viewers.

Recommendations

The recommendations summarized in Table 3 are drawn from this study’s findings and the broader literature, and are directly adapted from the World Health Organization’s 2019 guidelines “Preventing Suicide: A Resource for Filmmakers and Others Working on Stage and Screen” [13]. This alignment ensures that all guidance reflects practical, internationally recognized best practices for responsible media portrayals of suicide prevention and storytelling.

**Table 3:** Recommendations for responsible media representation of suicide.

For filmmakers and content creators	
Avoid sensationalism	Do not normalize, glamorize, or romanticize suicide. Avoid explicit details about methods.
Include content warnings	Clearly warn viewers about potentially distressing content.
Show help-seeking and recovery	Portray characters seeking help, using crisis resources, or recovering, to model positive behaviors, and show competent, strong characters.
Consult experts	Collaborate with mental health professionals during script development.
Follow guidelines	Adhere to established guidelines from the WHO.
For educators and mental health professionals	
Use media as teaching tools	Select films or documentaries that foster an understanding of suicide complexity.
Promote media literacy	Teach young people to critically evaluate media portrayals of suicide and mental health.
For policymakers and platforms	
Mandate warnings and resources	Require streaming platforms to include content warnings and links to crisis resources for media depicting suicide.
Support research	Fund further research into the effects of media portrayals on vulnerable populations and effective intervention strategies.

Limitations

Cohen’s kappa could not be calculated for eight items in the MoVIES scale. This limitation occurred due to a small sample size. Additionally, the scope of this study was intentionally kept narrow by focusing exclusively on films. Many television and streaming series were not included in order to remain consistent with the defined scope of the research.

## Conclusions

In summary, while creative freedom is vital, the entertainment industry has a responsibility to balance expression with the potential for harm. By fostering collaboration between creators and mental health experts, and adhering to evidence-based guidelines, the media can play a constructive role in suicide prevention, raising awareness, reducing stigma, and ultimately saving lives. The main strengths of this study include its systematic approach using validated quantitative and qualitative tools, robust inter-rater reliability measures, and an analysis that considered both risk and protective factors, as well as narrative themes. Future research should expand the scope to include television, streaming series, and broader cultural contexts, and investigate not only portrayals but also their effects on vulnerable audiences, as well as the effectiveness of interventions such as content warnings and mental health resources.
